# Low-dose CT coronary angiography for assessment of coronary artery disease in patients with type 2 diabetes - A cross-sectional study

**DOI:** 10.1186/s12872-015-0143-9

**Published:** 2015-11-14

**Authors:** Geir Reinvik Ulimoen, Anne Pernille Ofstad, Knut Endresen, Lars Gullestad, Odd Erik Johansen, Arne Borthne

**Affiliations:** Department of Radiology, Akershus University Hospital, PB 1000, 1478 Lorenskog, Norway; Department of Medical Research, Bærum Hospital, Vestre Viken Hospital Trust, 3004 Drammen, Norway; Department of Cardiology, Oslo University Hospital, Rikshospitalet, 0372 Oslo, Norway; University of Oslo, Oslo, Norway

**Keywords:** Type 2 diabetes mellitus, Cardiovascular complications, Imaging, CT angiography, Coronary artery calcification, Risk assessment

## Abstract

**Background:**

Silent coronary artery disease (CAD) is prevalent in type 2 diabetes mellitus (T2DM). Although coronary computed tomography angiography (CCTA) over recent years has emerged a useful tool for assessing and diagnosing CAD it’s role and applicability for patients with T2DM is still unclarified, in particular in asymptomatic patients. We aimed to assess the role of CCTA in detecting and characterizing CAD in patients with T2DM without cardiac symptoms when compared to gold standard invasive coronary angiography (ICA).

**Methods:**

This was a cross-sectional analysis of patients with T2DM without symptomatic CAD enrolled in the Asker and Baerum Cardiovascular Diabetes Study who, following clinical examination and laboratory assessment, underwent subsequently CCTA and ICA.

**Results:**

In total 48 Caucasian patients with T2DM (36 men, age 64.0 ± 7.3 years, diabetes duration 14.6 ± 6.4 years, HbA1c 7.4 ± 1.1 %, BMI 29.6 ± 4.3 kg/m^2^) consented to, and underwent, both procedures (CCTA and ICA). The population was at intermediate cardiovascular risk (mean coronary artery calcium score 269, 75 % treated with antihypertensive therapy). ICA identified a prevalence of silent CAD at 17 % whereas CCTA 35 %. CCTA had a high sensitivity (100 %) and a high negative predictive value (100 %) for detection of patients with CAD when compared to ICA, but the positive predictive value was low (47 %).

**Conclusions:**

Low-dose CCTA is a reliable method for detection and exclusion of significant CAD in T2DM and thus may be a useful tool for the clinicians. However, a low positive predictive value may limit its usefulness as a screening tool for all CAD asymptomatic patients with T2DM. Further studies should assess the applicability for risk assessment beyond the evaluation of the vascular bed.

## Background

Type 2 diabetes mellitus (T2DM) is a chronic metabolic disease that represents a major public health concern. T2DM affects more than 382 million people world-wide and the prevalence is expected to increase substantially [1]. Cardiovascular (CV) disease is the most common cause of death in patients with T2DM [2, 3]. The prevalence of undiagnosed coronary artery disease (CAD) among asymptomatic patients with T2DM is high and independent assessments using different diagnostic techniques, i.e., invasive coronary angiography (ICA) [4] or maximum stress-test [5] have found that more than 1 in 5 adults with T2DM have significant CAD. The diagnosis of CAD may be missed or delayed in these patients since the typical symptoms of CAD are often absent in patients with long-standing T2DM, which in turn further increases their risk for CV events. To potentially prevent CV events it may therefore be important to detect subclinical CAD in T2DM to enable appropriate intervention to reduce the risk of fatal and non-fatal cardiac events.

Findings to date to not support widespread screening for CAD in patients with T2DM, but this may partly be related to lack of a reliable and cost-effective screening tool [6].

ICA has been the accepted gold standard method for assessing the presence, localization, and severity of CAD, but has a substantial procedural cost and is an invasive method associated with a risk for complications. Coronary computed tomography angiography (CCTA) has over recent years emerged as an alternative to ICA for CAD assessment and the 64-slice generation scanners multidetector computer tomography (MDCT) is now considered to have high diagnostic performance for detection of significant coronary stenosis [7] in different populations. So far only few studies have assessed the role of MDCT in patients with T2DM [8, 9], and its full potential is not fully understood, in particular in light of studies being suggestive of a reduced diagnostic performance of MDCT compared to non-T2DM populations [10]. Thus, the role of CCTA is considered uncertain in asymptomatic high-risk patients like patients with T2DM [11].

In this study we aimed to assess the role of CCTA in detecting and characterizing CAD in patients with T2DM without cardiac symptoms. To provide robustness of using CCTA, all patients underwent both CCTA and gold standard ICA, and to our knowledge this is the first study to employ both strategies in asymptomatic patients with T2DM.

## Methods

### Study population

Caucasian subjects with T2DM without cardiac symptoms who were enrolled in the Asker and Baerum Cardiovascular Diabetes (ABCD) study in 2002-2004 [12] and still alive, were eligible for participation in this 7-year cross-sectional follow-up study (ABCD-2). Classification of subjects as being asymptomatic was based on patient history and clinical assessment (i.e., free from cardio-pulmonary symptoms) [13]. In addition to T2DM, diagnosed in accordance with diagnostic criteria by the World Health Organization [14], the inclusion criteria in the ABCD-study were age 18-75 years and at least one additional CV risk factor (hypertension [treated or 24 h systolic/diastolic blood pressure ≥140/90 mmHg], dyslipidaemia [treated or total cholesterol ≥ 5.0 mmol/L, HDL-cholesterol ≤1.0 mmol/L in men, or ≤1.1 mmol/L in women or triglycerides ≥2.0 mmol/L], past or prior smoking, premature CAD in 1^st^ degree family [male < 55 years, female < 65 years], or microalbuminuria). Exclusion criteria in this imaging follow-up study were irregular heart rate, pregnancy, known allergy to iodinated contrast medium and elevated serum creatinine (female > 120 μmol/L, male > 130 μmol/L).

The study complied with the Declaration of Helsinki and was approved by the Regional Committee for Medical and Health Research Ethics. All participating patients gave written informed consent.

### Investigation and study procedures

Following an assessment of medical history, clinical examination including cardiopulmonary exercise testing and laboratory assessment, patients were voluntarily referred for subsequently assessments with CCTA or ICA regardless of the results of the non-invasive tests. Both CAD imaging modalities had to be completed within a 6 month time window by experienced radiologists or cardiologists.

#### Cardiopulmonary exercise test

Exercise induced cardiac ischemia was assessed using a modified conventional maximum symptom-limited 1-min incremental cardiopulmonary exercise test on a cycle ergometer (Siemens-Elema, Germany). Cycling frequency had to be at least 60 rounds pr minute. The criterion for classifying a test as positive was the traditional ≥1 mm ST-segment depression observed in two adjacent leads.

#### Laboratory assessment

Peripheral venous blood samples were drawn in the morning after an overnight fast and routine laboratory parameters were immediately analyzed by the local laboratory. For all assays, the intra-and inter assay coefficients of variation were <10 %.

#### Coronary Computed Tomography Angiography (CCTA)

CCTA was performed at Akershus University Hospital, Lørenskog, Norway, using a 256-slice scanner (Brilliance iCT, Philips Healthcare, Cleveland, OH, USA). Metoprolol was intravenously administered with a titration dose up to 15 mg in all patients with heart rate >70 bpm. A low-dose scout image followed by unenhanced prospectively ECG-triggered coronary artery calcium (CAC) scoring acquisition. Imaging parameters includes X-ray tube voltage 120 kV, detector configuration 32 × 0.625 mm, gantry rotation time 0.33 s, field of view 250 mm, reconstructed slice thickness 2.5 mm and increment 2.5 mm. Angiographic acquisition was performed using a prospectively ECG-triggered step-and-shoot mode and the contrast medium iomeprol (Iomeron 400 mg/ml, Bracco Imaging SpA). A detector configuration of 128 × 0.625 mm with dual z-focal spot positions was used. Gantry rotation time was 0.27 s, temporal resolution 135 msec and X-ray tube voltage was 120 kV.

Both calcium scoring acquisition and angiographic acquisition were started at a predefined time point at 75 % of the RR interval corresponding to a physiological phase of ventricular diastasis, with the tube output turned off during the other phases of the RR interval. The effective radiation dose of the CT exam was estimated by the product of the dose length product from the dose report of the CT scanner and a conversion coefficient for the chest (k = 0.014 mSv*mGy − 1*cm − 1) [15].

Analysis was performed by two experienced radiologist in consensus, using specialized software on a dedicated workstation (Comprehensive Cardiac Analysis, Extended Brilliance Workspace V4.02, Philips Healthcare, Cleveland, OH, USA) including semi-automated software for analysis of Agatston calcium score [16]. The interpreters were blinded for the results of ICA and non-invasive tests. For analysis, the coronary arteries were segmented into a 15-segment American Heart Association (AHA) model [17] and segments with a luminal diameter ≥1 mm were evaluated. Image quality was ranked using a 4-point scale (1: good image quality, vessels with sharp edges without discontinuity and artifacts; 2: adequate image quality, vessels with minor artifacts or slight blurring; 3: decreased image quality, evaluation difficult but still possible; 4: non-interpretable). Calcifications were ranked using a 3-point scale (1: no calcifications; 2: minor calcifications; 3: severe calcifications). All interpretable segments were scored with a 4-point scale (0: no stenosis or ≤24 % lumen reduction; 1: 25-49 % stenosis; 2; 50-74 % stenosis; 3: ≥75 % stenosis). A stenosis of ≥50 % was considered significant.

#### Invasive coronary angiography

ICA was performed by radial or femoral approach according to the standard Judkins technique using 6 F diagnostic catheters (Cordis Corporation, Miami, FL, USA) and the contrast medium iodixanol (Visipaque 320 mg/ml, Amersham Health, GE Healthcare AS, Oslo, Norway). The angiograms were performed at the catheterization lab at Oslo University Hospital Rikshospitalet, Oslo, Norway and analyzed by an experienced cardiologist who was blinded to the results of non-invasive tests and CCTA. All angiograms were evaluated by visual analysis supported by a quantitative coronary analysis (QCA) program (Sectra Cardiology Package 1.0, Sectra Imtec, Sweden) with similar segment model and stenosis grading as for CCTA.

### Statistics

Data are reported as frequencies or mean with standard deviation. Correlations analysis between calcium scores and baseline HbA1c and duration of T2DM was performed (Spearman’s correlation). Cohen’s kappa statistics were used to describe concordance between the methods interpreted by the guidelines of Landis and Koch [18]. Sensitivity, specificity, and positive and negative predictive values with corresponding 95 % confidence intervals (CI) were calculated for the detection of significant (>50 %) coronary artery stenoses with CCTA, using ICA as the gold standard and Vassarstats (Vassar College, Poughkeepsie, N.Y., USA) for statistical analysis. For all other statistical analysis, IBM SPSS Version 22 was used.

## Results

### Demographics

Of 93 patients consenting to participate in ABCD-2, 56 patients had no contraindications to the imaging procedures and consented to perform both CCTA and ICA. In eight patients CT angiography was not performed, because of calcium score >1000, rendering 48 patients eligible for analysis. The patient population studied was obese (mean BMI 29.6 kg/m2) and middle-aged (mean age 64 years) with a long history of T2DM (mean duration 15.6 years), not at optimal glycemic control (mean HbA1c 7.4 %). The majority were males (75 %). Further characteristics are given in Table 1.

**Table 1:** Baseline characteristics of the study participants

Number	48
Background information	
Gender (male, *n* (%)	36 (75 %)
Age (years), mean ± SD (range)	64.0 ± 7.3 (41.4–76,7)
Body Mass Index (kg/m^2^), mean ± SD (range)	29.6 ± 4.3 (21.7–39.7)
T2DM duration (years), mean ± SD (range)	14.6 ± 6.4 (6.8–37.7)
Medical history and medications	
History of smoking, *n* (%)	19 (39.6 %)
Family history of premature CAD (1^st^ degree relative), *n* (%)	24 (50 %)
Any medication for hypertension, *n* (%)	36 (75 %)
Any lipidlowering medication, *n* (%)	38 (79 %)
Any blood glucose lowering medication, *n* (%)	46 (96 %)
-Any use of insulin, *n* (%)	9 (18.7 %)
Laboratory and clinically assessment	
HbA1c (%), mean ± SD	7,4 ± 1.1
Serum creatinine (μmol/L), mean ± SD (range)	71.4 ± 16.7 (42–117)
Total cholesterol (mmol/L), mean ± SD	4.3 ± 1.0
LDL cholesterol (mmol/L), mean ± SD	2.4 ± 0.8
HDL cholesterol (mmol/L), mean ± SD	1.4 ± 0.4
Triglycerides (mmol/L), mean ± SD	1.3 ± 0.5
Systolic blood pressure (mmHg), mean ± SD	138 ± 17
Diastolic blood pressure (mmHg), mean ± SD	79 ± 8
Heart rate (bpm), mean ± SD	73 ± 13
Exercise ECG	
- Positive, *n* (%) - Inconclusive, *n* (%) - Negative, *n* (%)	4 (8.3 %) 11 (22.9 %) 33 (68.8 %)

### Imaging assessments

In total 99 % (588 out of 594) of coronary segments with a luminal diameter ≥1 mm from the full patient population were eligible for analysis with CCTA. Only 6 segments were non-interpretable, mainly because of severe artifacts from pacemaker electrodes. Mean Agatston score was 269, but ranged from 0 to 976 and this did not correlate with neither diabetes duration (r = 0.05, p = 0.75) or HbA1c (r = -0.14, p = 0.35). Image quality and degree of calcifications are detailed in Table 2. In analyzing the 588 segments, CCTA had a sensitivity of 90 % (9 of 10) and a specificity of 96 % (557 of 578) for detection of coronary stenosis ≥50 % using ICA as gold standard (Table 3). The positive predictive value was 30 % (9 of 30) and the negative predictive value 99 % (556 of 557). Degree of stenosis per segment analysis and maximum degree of any stenosis per patient analysis are given in Table 3. CCTA wrongly classified 21 segments (3.8 %) as having significant stenosis. Of these 21 segments ICA classified 10 segments with 25-49 % stenosis and the remaining 11 segments without any stenosis (≤24 % lumen reduction). All these 21 segments were found to have either severe calcifications or a combination of calcifications and minor artifacts on CCTA.

**Table 2:** Coronary CTA, scan characteristics and results

Number of patients, *n*	48
Intravenous β-blocker given, *n* (%)	18 (37.5 %)
Heart rate during scan, mean ± SD (range)	63.7 ± 7.6 (49–88)
Radiation dose (mSv), mean ± SD (range)	3.8 ± 0.7 (2.5–5.0)
Agatston score, mean ± SD (range)	269.0 ± 292.8 (0–976)
Image quality per segment, *n* (%)	
1. Good 2. Adequate 3. Decreased	420 (71 %) 132 (23 %) 36 (6 %)
Degree of calcifications per segment, *n* (%)	
1. None 2. Minor 3. Severe	374 (64 %) 171 (29 %) 43 (7 %)

**Table 3:** Results of CCTA and ICA

Diagnostic accuracy of CCTA using ICA as gold standard	Per segment	Per patient
- Sensitivity, % (CI) - Spesificity, % (CI) - Positive predictice value, % (CI) - Negative predictive value, % (CI)	90 % (54–99) 96 % (94–98) 30 % (15–50) 99 % (99–100)	100 % (60–100) 78 % (61–89) 47 % (24–72) 100 % (86–100)
Degree of stenosis *n* (%), per segment analysis	CCTA	ICA
0. No stenosis or ≤24 % lumen reduction 1. 25–49 % stenosis 2. 50–74 % stenosis 3. ≥75 % stenosis	447 (76.0 %) 110 (18.7 %) 29 (4.9 %) 2 (0.4 %)	553 (94.0 %) 25 (4.3 %) 9 (1.5 %) 1 (0.2 %)
Maximum degree of any stenosis, *n* (%), per patient analysis	CCTA	ICA
0. No stenosis or ≤24 % lumen reduction 1. 25–49 % stenosis 2. 50–74 % stenosis 3. ≥75 % stenosis	10 (20.8 %) 21 (43.8 %) 15 (31.3 %) 2 (4.2 %)	28 (58.3 %) 12 (25.0 %) 7 (14.6 %) 1 (2.1 %)

In a per patient analysis, 17 % (8 of 48) had at least one stenosis ≥50 % in any segment at ICA. CCTA correctly identified all these patients, corresponding to a sensitivity of 100 %. Specificity was 78 %, positive predictive value 47 %, and negative predictive value 100 %. Estimated Kappa was 0,53, giving moderate agreement between the methods.

At the maximum exercise test, the mean achieved work capacity was 143 ± 44 Watts. 15 patients (31 %) had signs of silent ischemia with positive or inconclusive test. When comparing silent ischemia as detected on the maximum exercise test versus CCTA and ICA estimated Kappa were 0,16 and 0,17 respectively, yielding poor agreement.

## Discussion

This study assessed the applicability of using a modern non-invasive imaging modality to detect significant CAD in an intermediate CV risk cohort of patients with T2DM and compared its results with the gold standard for assessing this, namely ICA. This study, to the best of our knowledge, is the first that have screened asymptomatic patients with both CCTA and ICA. The main finding of our study was that 256-slice CCTA with step-and-shoot technique provides excellent sensitivity and yields a high negative predictive value for excluding significant CAD in patients with T2DM, whereas the specificity and positive predictive value were lower (78 % and 47 %, respectively). These data are in agreement with results of other assessments on sensitivities in patients with low degree of vasculopathy, i.e. low calcium scores [19]. Our study also shows that the technique provides good feasibility with nearly all coronary segments eligible for analysis. Radiation dose is lower than with earlier generation CT scanners [20] and may be further reduced with newer techniques like iterative reconstruction [21, 22]. Our data suggested that despite a mean T2D duration of 15 years, mean Agatston score was 269, indicating that the population still was at intermediate CV risk, and not too advanced, as one could have expected based on the lengthy diabetes duration [23].

CCTA is well known to yield many false positives, but the low positive predictive values in our study seems inferior to most other reports although the results are varying [24]. There may be several contributions to this. Positive and negative predictive values are generally influenced by prevalence of disease, and the low prevalence of significant CAD in this study would contribute to lower positive predictive value than in studies with higher prevalence. The coronary plaque burden and coronary calcium score are generally higher in diabetic than in non-diabetic patients [19, 25] and since calcified plaques can lead to overestimation of lesion severity [26] this may contribute to lower positive predictive value for CCTA in a diabetic population than in a non-diabetic population. Some studies have shown smaller coronary vessel calibre in patients with T2DM as compared to those without [27, 28], and this may affect the diagnostic accuracy of CCTA. Reduced diagnostic performance of CCTA in patients with T2DM is reported in the only known study comparing with patients without T2DM [10], but this study included mainly symptomatic patients and the prevalence of obstructive CAD was high. On the other side, a mean CAC value of 269 and 18 % patients with zero CAC score would also render CCTA a suboptimal test to rule out obstructive CAD among symptomatic patients [19].

The major established indications for use of CCTA are within groups of symptomatic patients with low or intermediate pretest probability of obstructive CAD, and in some pre- and postoperative settings [11]. A recent meta-analysis supports the use of CCTA as a first imaging test for low- and intermediate-risk patients presenting to the emergency department with chest pain [29]. The role of CCTA remains uncertain in asymptomatic high-risk patients like patients with T2DM, and even if there are reports finding a prognostic value of CCTA [8, 9] there is another recent study reporting that use of CCTA to screen asymptomatic patients with T2DM do not improve clinical outcome [30]. One study in patients with T2DM and mild anginal complaints demonstrated a crucial impact of ischemia on cardiac event rate and showed a prognostic value of myocardial perfusion scintigraphy (MPS [31], and a possible strategy may be to perform supplementary MPS in asymptomatic patients with positive CCTA. Another possible future improvement of screening CCTA may be a combination with computed tomography myocardial perfusion imaging, as there are recent promising reports about this method [32, 33], or to combine results of CCTA with prognostic biomarkers [34].

The relatively small number of included patients is a limitation of the generalizability of the study results, including the relatively low proportion of female patients.

## Conclusions

Low-dose CCTA provides excellent sensitivity and negative predictive value in detecting and excluding significant CAD in patients with T2DM without known or suspected CAD. The usefulness of this method as a screening tool in asymptomatic intermediate risk patients like in T2DM may be limited by a rather low positive predictive value.

## Abbreviations

CAC: coronary artery calcium.
CAD: coronary artery disease
CCTA: coronary computed tomography angiography
CV: cardiovascular
ICA: invasive coronary angiography
MDCT: multidetector computer tomography
T2DM: type 2 diabetes mellitus

## References

[1] IDF Diabetes Atlas. In*.*, 6th edn. Brussels, Belgium:International Diabetes Federation; 2013. Available from: www.idf.org/diabetesatlas

[2] Lorber D (2014). Importance of cardiovascular disease risk management in patients with type 2 diabetes mellitus. Diabetes Metab Syndr Obes.

[3] Stamler J, Vaccaro O, Neaton JD, Wentworth D (1993). Diabetes, other risk factors, and 12-yr cardiovascular mortality for men screened in the Multiple Risk Factor Intervention Trial. Diabetes Care.

[4] Johansen OE, Birkeland KI, Orvik E, Flesland O, Wergeland R, Ueland T, Smith C, Endresen K, Aukrust P, Gullestad L (2007). Inflammation and coronary angiography in asymptomatic type 2 diabetic subjects. Scand J Clin Lab Invest.

[5] Wackers FJ, Young LH, Inzucchi SE, Chyun DA, Davey JA, Barrett EJ, Taillefer R, Wittlin SD, Heller GV, Filipchuk N (2004). Detection of silent myocardial ischemia in asymptomatic diabetic subjects: the DIAD study. Diabetes Care.

[6] Upchurch CT, Barrett EJ (2012). Clinical review: Screening for coronary artery disease in type 2 diabetes. J Clin Endocrinol Metab.

[7] Janne d'Othee B, Siebert U, Cury R, Jadvar H, Dunn EJ, Hoffmann U (2008). A systematic review on diagnostic accuracy of CT-based detection of significant coronary artery disease. Eur J Radiol.

[8] Cademartiri F, Seitun S, Romano M, Maffei E, Fusaro M, Palumbo A, Aldrovandi A, Messalli G, Tresoldi S, Malago R (2008). Prognostic value of 64-slice coronary angiography in diabetes mellitus patients with known or suspected coronary artery disease compared with a nondiabetic population. Radiol Med.

[9] Van Werkhoven JM, Cademartiri F, Seitun S, Maffei E, Palumbo A, Martini C, Tarantini G, Kroft LJ, de Roos A, Weustink AC (2010). Diabetes: prognostic value of CT coronary angiography--comparison with a nondiabetic population. Radiology.

[10] Andreini D, Pontone G, Bartorelli AL, Agostoni P, Mushtaq S, Antonioli L, Cortinovis S, Canestrari M, Annoni A, Ballerini G (2010). Comparison of the diagnostic performance of 64-slice computed tomography coronary angiography in diabetic and non-diabetic patients with suspected coronary artery disease. Cardiovasc Diabetol.

[11] Taylor AJ, Cerqueira M, Hodgson JM, Mark D, Min J, O'Gara P, Rubin GD, Kramer CM, Berman D, Brown A (2010). ACCF/SCCT/ACR/AHA/ASE/ASNC/NASCI/SCAI/SCMR 2010 appropriate use criteria for cardiac computed tomography. A report of the American College of Cardiology Foundation Appropriate Use Criteria Task Force, the Society of Cardiovascular Computed Tomography, the American College of Radiology, the American Heart Association, the American Society of Echocardiography, the American Society of Nuclear Cardiology, the North American Society for Cardiovascular Imaging, the Society for Cardiovascular Angiography and Interventions, and the Society for Cardiovascular Magnetic Resonance. J Am Coll Cardiol.

[12] Johansen OE, Gullestad L, Blaasaas KG, Orvik E, Birkeland KI (2007). Effects of structured hospital-based care compared with standard care for Type 2 diabetes-The Asker and Baerum Cardiovascular Diabetes Study, a randomized trial. Diabet. Med..

[13] Gibbons RJ, Chatterjee K, Daley J, Douglas JS, Fihn SD, Gardin JM, Grunwald MA, Levy D, Lytle BW, O'Rourke RA (1999). ACC/AHA/ACP-ASIM guidelines for the management of patients with chronic stable angina: a report of the American College of Cardiology/American Heart Association Task Force on Practice Guidelines (Committee on Management of Patients With Chronic Stable Angina). J Am Coll Cardiol.

[14] World Health Organization (1999). Definition, diagnosis and classification of diabetes mellitus and its complications - Part 1: Diagnosis and Classification of Diabetes Mellitus.

[15] Halliburton SS, Abbara S, Chen MY, Gentry R, Mahesh M, Raff GL, Shaw LJ, Hausleiter J (2011). SCCT guidelines on radiation dose and dose-optimization strategies in cardiovascular CT. J Cardiovasc Comput Tomogr.

[16] Agatston AS, Janowitz WR, Hildner FJ, Zusmer NR, Viamonte M, Detrano R (1990). Quantification of coronary artery calcium using ultrafast computed tomography. J Am Coll Cardiol.

[17] Austen WG, Edwards JE, Frye RL, Gensini GG, Gott VL, Griffith LS, McGoon DC, Murphy ML, Roe BB (1975). A reporting system on patients evaluated for coronary artery disease. Report of the Ad Hoc Committee for Grading of Coronary Artery Disease, Council on Cardiovascular Surgery, American Heart Association. Circulation.

[18] Landis JR, Koch GG (1977). The measurement of observer agreement for categorical data. Biometrics.

[19] Youssef G, Budoff MJ (2012). Coronary artery calcium scoring, what is answered and what questions remain. Cardiovasc. Diagn Ther.

[20] Hou Y, Yue Y, Guo W, Feng G, Yu T, Li G, Vembar M, Olszewski ME, Guo Q (2012). Prospectively versus retrospectively ECG-gated 256-slice coronary CT angiography: image quality and radiation dose over expanded heart rates. Int J Cardiovasc Imaging.

[21] Carrascosa P, Rodriguez-Granillo GA, Capunay C, Deviggiano A (2013). Low-dose CT coronary angiography using iterative reconstruction with a 256-slice CT scanner. World J. Cardiol..

[22] Hou Y, Ma Y, Fan W, Wang Y, Yu M, Vembar M, et al. Diagnostic accuracy of low-dose 256-slice multi-detector coronary CT angiography using iterative reconstruction in patients with suspected coronary artery disease. Eur. Radiol. 2014;24(1):3-11. 10.1007/s00330-013-2969-9 23887663

[23] Greenland P, Bonow RO, Brundage BH, Budoff MJ, Eisenberg MJ, Grundy SM, Lauer MS, Post WS, Raggi P, Redberg RF (2007). ACCF/AHA 2007 clinical expert consensus document on coronary artery calcium scoring by computed tomography in global cardiovascular risk assessment and in evaluation of patients with chest pain: a report of the American College of Cardiology Foundation Clinical Expert Consensus Task Force (ACCF/AHA Writing Committee to Update the 2000 Expert Consensus Document on Electron Beam Computed Tomography) developed in collaboration with the Society of Atherosclerosis Imaging and Prevention and the Society of Cardiovascular Computed Tomography. J Am Coll Cardiol.

[24] Jiang B, Wang J, Lv X, Cai W (2014). Dual-source CT versus single-source 64-section CT angiography for coronary artery disease: A meta-analysis. Clin Radiol.

[25] Maffei E, Seitun S, Nieman K, Martini C, Guaricci AI, Tedeschi C, Weustink AC, Mollet NR, Berti E, Grilli R (2011). Assessment of coronary artery disease and calcified coronary plaque burden by computed tomography in patients with and without diabetes mellitus. Eur Radiol.

[26] Leber AW, Knez A, von Ziegler F, Becker A, Nikolaou K, Paul S, Wintersperger B, Reiser M, Becker CR, Steinbeck G (2005). Quantification of obstructive and nonobstructive coronary lesions by 64-slice computed tomography: a comparative study with quantitative coronary angiography and intravascular ultrasound. J Am Coll Cardiol.

[27] Mosseri M, Nahir M, Rozenman Y, Lotan C, Admon D, Raz I, Gotsman MS (1998). Diffuse narrowing of coronary arteries in diabetic patients: the earliest phase of coronary artery disease. Cardiology.

[28] Elezi S, Kastrati A, Pache J, Wehinger A, Hadamitzky M, Dirschinger J, Neumann FJ, Schomig A (1998). Diabetes mellitus and the clinical and angiographic outcome after coronary stent placement. J Am Coll Cardiol.

[29] D'Ascenzo F, Cerrato E, Biondi-Zoccai G, Omede P, Sciuto F, Presutti DG, Quadri G, Raff GL, Goldstein JA, Litt H (2013). Coronary computed tomographic angiography for detection of coronary artery disease in patients presenting to the emergency department with chest pain: a meta-analysis of randomized clinical trials. Eur Heart J. Card. Imaging.

[30] Muhlestein JB, Lappe DL, Lima JA, Rosen BD, May HT, Knight S, Bluemke DA, Towner SR, Le V, Bair TL (2014). Effect of screening for coronary artery disease using CT angiography on mortality and cardiac events in high-risk patients with diabetes: the FACTOR-64 randomized clinical trial. JAMA.

[31] Wiersma JJ, Verberne HJ, ten Holt WL, Radder IM, Dijksman LM, van Eck-Smit BL, Trip MD, Tijssen JG, Piek JJ (2009). Prognostic value of myocardial perfusion scintigraphy in type 2 diabetic patients with mild, stable angina pectoris. J. Nucl. Cardiol..

[32] George RT, Mehra VC, Chen MY, Kitagawa K, Arbab-Zadeh A, Miller JM, Matheson MB, Vavere AL, Kofoed KF, Rochitte CE (2014). Myocardial CT perfusion imaging and SPECT for the diagnosis of coronary artery disease: a head-to-head comparison from the CORE320 multicenter diagnostic performance study. Radiology.

[33] Magalhaes TA, Kishi S, George RT, Arbab-Zadeh A, Vavere AL, Cox C, Matheson MB, Miller JM, Brinker J, Di Carli M (2015). Combined coronary angiography and myocardial perfusion by computed tomography in the identification of flow-limiting stenosis - The CORE320 study: An integrated analysis of CT coronary angiography and myocardial perfusion. J Cardiovasc Comput Tomogr.

[34] Ofstad AP, Gullestad L, Orvik E, Aakhus S, Endresen K, Ueland T, Aukrust P, Fagerland MW, Birkeland KI, Johansen OE (2013). Interleukin-6 and activin A are independently associated with cardiovascular events and mortality in type 2 diabetes: the prospective Asker and Baerum Cardiovascular Diabetes (ABCD) cohort study. Cardiovasc Diabetol.

